# SPECT/CT to quantify early small airway disease and its relationship to clinical symptoms in smokers with normal lung function: a pilot study

**DOI:** 10.3389/fphys.2024.1417463

**Published:** 2024-08-15

**Authors:** Daniel Juneau, Antoine Leblond, Rami Chatta, Valérie Lévesque, Annabelle Lussier, Bruno-Pierre Dubé

**Affiliations:** ^1^ Département d’imagerie Médicale, Service de Médecine Nucléaire, Centre Hospitalier de l’Université de Montréal (CHUM), Montréal, QC, Canada; ^2^ Centre de Recherche du Centre Hospitalier de l’Université de Montréal (CRCHUM), Montréal, QC, Canada; ^3^ Faculté de médecine, Université de Montréal, Montréal, QC, Canada; ^4^ Département de Médecine, Service de Pneumologie, Centre Hospitalier de l’Université de Montréal (CHUM), Montréal, QC, Canada

**Keywords:** small airway, ventilatory distributions, SPECTCT, tobacco, pulmonary function test

## Abstract

**Introduction:**

Smokers frequently display respiratory symptoms despite the fact that their pulmonary function tests (PFTs) can be normal. Quantitative lung ventilation single-photon emission computed tomography (SPECT/CT) can provide a quantification of lung ventilatory homogeneity and could prove useful as an early marker of airway disease in smokers. We measured the effects of smoking on regional ventilation distribution in subjects with normal lung function and evaluated whether ventilation distribution in these subjects is related to lung function tests results and clinical symptoms.

**Methods:**

Subjects without any history of respiratory disease were prospectively recruited and separated in two groups: active smokers (AS: ≥10 cigarettes/day and history of ≥15 pack-years) and never smokers (NS: lifetime exposure of <5 cigarettes). All subjects performed PFTs (which had to be normal, defined as z-score values of forced expiratory volume in 1 s (FEV1), forced vital capacity (FVC), FEV1/FVC ratio, total lung capacity (TLC) residual volume and diffusion capacity (DLCO) all falling between −1.65 and +1.65) and underwent SPECT/CT with Technegas, which generated subject- specific ventilation heterogeneity maps. The area under the compensated coefficient of variation (CV) density curve for CV values > 40%, (AUC-CV40%) was used as the measure of ventilation heterogeneity.

**Results:**

30 subjects were recruited (15 per group). Subjects in the AS group displayed higher dyspnea levels (1 [1–2] vs. 0 [0–1] units on mMRC scale, *p* < 0.001). AUC- CV40% was significantly higher in the AS group (0.386 ± 0.106 vs. 0.293 ± 0.069, *p* = 0.004). AUC-CV40% was significantly correlated to FEV1 (rho = −0.47, *p* = 0.009), DLCO (rho = −0.49, *p* = 0.006), CAT score (rho = 0.55, *p* = 0.002) and mMRC score (rho = 0.54, *p* = 0.002). Subjects with mMRC >0 had higher AUC-CV40% values than those without dyspnea (0.289 ± 0.071 vs. 0.378 ± 0.102, *p* = 0.006), while FEV1 and DLCO were not different between those groups. ROC analyses showed that the AUC for AUC-CV40% in identifying subjects with mMRC score >0 was 0.78 (95%CI 0.61–0.95, *p* = 0.009), which was significantly higher than that of FEV1 and DLCO.

**Discussion:**

In smokers with normal lung function, ventilatory inhomogeneities can be quantified using SPECT/CT. AUC-CV40% values are related to lung function decline and to respiratory symptomatology, suggesting a potential role for this marker in the evaluation of symptomatic smokers.

## Introduction

Ventilation heterogeneity is a hallmark feature of most obstructive pulmonary diseases. Chronic obstructive pulmonary disease (COPD) is pathologically and physiologically characterized by small airway destruction and marked airway cellular inflammation, which result in prominent expiratory airflow limitation, air trapping, hyperinflation and abnormal gas exchange (Bourdin et al., 2009). COPD is strongly linked to exposition to inhaled irritants, most notably tobacco smoke, and as such is a potentially preventable disease. COPD-related morbidity, mortality and social costs are high: in Canada, COPD is the main cause of hospital admission among all chronic diseases and is the fourth leading cause of death (Mannino and Buist, 2007; Mittmann et al., 2008).

The diagnosis of COPD requires the objective demonstration of persistent expiratory airflow limitation using spirometry (Global Initiative for Chronic Obstructive Lung Disease). In the right clinical context, a post-bronchodilator forced expiratory volume in 1 s (FEV_1_)/forced vital capacity (FVC) ratio <0.70 is considered indicative of the presence of COPD. However, the natural history of COPD represents a slowly progressive continuum: active smokers that do not meet the criteria for COPD are still at risk of developing the disease (Brito-Mutunayagam et al., 2010; Woodruff et al., 2016; Elbehairy et al., 2017). In fact, when compared to healthy non-smokers, active smokers can already show some pathological and clinical features of the disease (Brito-Mutunayagam et al., 2010; Regan et al., 2015; Woodruff et al., 2016), even in the absence of overt spirometry-confirmed COPD. Most notably, these patients report increased levels of resting dyspnea, chronic cough, lower exercise capacity, exercise-induced dynamic hyperinflation and marked airway inflammatory cellular infiltration, while conserving normal pulmonary function test values (Stavem et al., 2006; Tsushima et al., 2006; Brito-Mutunayagam et al., 2010; Regan et al., 2015; Woodruff et al., 2016; Bhatt et al., 2017; Bodduluri et al., 2017; Elbehairy et al., 2017). These findings highlight the negative, clinically measurable effects of tobacco smoking on pulmonary function and clinical symptoms, but also the limitations of standard pulmonary function testing in identifying the presence of early, mild airway disease and quantifying physiological limitations in these subjects (Elbehairy et al., 2017). Although more specialized pulmonary lung function testing techniques such as the use of forced oscillometry and the measurement of lung clearance index can provide insight on more subtle airway disease compared with spirometry (Horsley, 2009; Ram et al., 2019), these techniques are infrequently available in clinical practice.

Quantitative lung ventilation single-photon emission computed tomography (SPECT/CT) allows an objective quantification of the regional heterogeneity of ventilation in humans (Sando et al., 1997; Nagao et al., 2000; Petersson et al., 2009; Suga et al., 2010; Jogi et al., 2011; Ax et al., 2013; Norberg et al., 2013; Norberg et al., 2014). The coefficient of variation (CV) of the distribution of a radiotracer, inhaled during the test, allows the generation of heterogeneity maps and density curves of small elements of the lung (Norberg et al., 2013; Norberg et al., 2014). The area under the curve of a pre-determined threshold for CV values (AUC-CV) is a marker of ventilation (in)homogeneity and could be of value in the clinical evaluation of the severity and distribution of lung disease. AUC-CV values have been shown to be sensitive to the presence of COPD, asthma, air trapping and are correlated to even slight anomalies in pulmonary function testing (PFT) in otherwise healthy subjects (Nagao et al., 2000; Suga et al., 2010; Ax et al., 2013; Norberg et al., 2013; Norberg et al., 2014). As such, the measurement of AUC-CV values could prove useful as an early marker of airway disease in active smokers at risk of developing COPD and could provide physicians with valuable information regarding the state of the respiratory system in these patients, but its use in this context has never been formally tested. We therefore designed this study to explore the question of whether ventilation SPECT/CT could provide clinically relevant information on airway disease in active smokers without overt lung disease on standard lung function testing. More specifically, the objectives of the study are: 1) To quantify the effects of active smoking on regional ventilation distribution in subjects with normal lung function testing and 2) to evaluate whether regional ventilation distribution in these subjects is related to underlying lung function tests results and clinical symptoms.

## Methods

### Study participants

This is a pilot study in which subjects were prospectively recruited at the pulmonary function laboratory of the Centre Hospitalier de l’Université de Montréal between July 2018 and March 2022. Patients were included in two mutually exclusive groups according to smoking status: never smokers (NS) and active smokers (AS).

For the AS group, inclusion criteria were: age ≥ 18 years; normal pulmonary lung function testing on the day of inclusion [defined as the presence of all of the following: FEV_1_/FVC ratio, FVC, FEV_1_, total lung capacity (TLC), residual volume (RV), functional residual capacity (FRC), inspiratory capacity (IC), RV/TLC ratio and diffusion capacity of the lung for carbon monoxide (DLCO) all within the lower and upper limits of normal (LLN and ULN, defined as z-scores values falling between −1.65 and 1.65) (Stanojevic et al., 2022), post-bronchodilator change in FEV_1_ and FVC (if performed) of < 6% and 100 mL]; body mass index (BMI) of < 40 kg/m^2^; active tobacco smoking of ≥ 10 cigarettes per day with a total smoking history of ≥ 15 pack-years.

For the NS group, inclusion criteria were the same as stated above except for the smoking history, which had to be reported as a lifetime history of <5 cigarettes or cigars and <5 days of using an electronic cigarette, with no smoking or vaping in the year before inclusion.

For both groups, exclusion criteria were: pregnancy; breastfeeding; any condition impeding reliable or complete pulmonary lung function testing (such as poor technique or claustrophobia limiting the performance of body plethysmography); any self-reported history or demonstrated diagnosis of COPD, asthma, venous thromboembolic disease, cystic fibrosis, bronchiectasis, interstitial lung disease, pulmonary hypertension, lung cancer, lung resection surgery, pneumothorax or pleural disease. In addition, the medical file of each potential subject was searched for the following diagnostic tests before inclusion, which if performed in the 5 years before inclusion had to show no significant abnormality (defined in parentheses): methacholine challenge test [provocative concentration (PC_20_) of methacholine of >16 mg/mL], chest radiograph or chest computed-tomography (absence of significant parenchymal, pleural, thoracic/ribcage or pulmonary vascular anomaly, as assessed by a radiologist with expertise in chest imagery). The ethical board of the CHUM approved the study (Project ID 18.149).

### Enrolment and intervention protocol

Patients undergoing routine, physician-ordered PFTs at the CHUM were prospectively screened by a member of the study team after the completion of the test. Patients meeting inclusion criteria and who provided informed, written consent were included in the study. Demographic data, indication for PFT, PFT results, comorbidities and clinical data were retrieved from medical files. Smoking history, dyspnea level [using the modified Medical Research Council scale (Bestall et al., 1999)] and respiratory symptoms [using the COPD Assessment Test–CAT (Jones et al., 2009)] were directly obtained from the subjects. A Technegas ventilation SPECT/CT was performed either immediately following the completion of the PFTs (for 28 out of 30 subjects) or within 48 h in a second visit (for 2 out of 30 subjects). There was no follow-up visit.

### Technegas ventilation SPECT/CT protocol and scan acquisition/reconstruction

Technegas was prepared with a Technegas Generator (Cyclomedica) according to the manufacturer recommendations with a simmer phase and a burning phase. 95% ethanol was used to wet the carbon crucible. The crucible was loaded with 20–30 mCi of Tc99m. Technegas was administered to the subjects within 10 min of its preparation, in a separate room than the scanning room. The inhalation technique was rehearsed with the subjects prior to the actual inhalation. Subjects were in the supine position and a mouthpiece and nose clip were used. Subjects were instructed to take 3 breaths of Technegas, starting after normal exhalation. A survey meter was used to monitor the quality of the inhalation. A lower threshold criterion of 4,500 counts per minute at the anterior chest surface was used. If the measured activity was lower than this threshold, the subjects were instructed to take additional breaths. This step was repeated until the lower threshold criterion was satisfied.

A Technegas ventilation SPECT/CT was acquired on a Discovery NM/CT 670 camera (GE Healthcare) with the following parameters for SPECT scan: LEHR collimator, energy window of 20% centered at 140 keV, zoom factor of 1.28, 128 × 128 matrix, 60 s/image, 30 images per camera head in step and shoot mode. For the CT scan, the parameters were 16 × 0.625 mm collimation, 120 kVp, 150 mA, rotation time of 0.5 s, pitch of 1.375:1, 512 × 512 matrix, FBP reconstruction, 1.25 mm slice thickness, soft reconstruction filter.

### SPECT/CT data processing

Images were reconstructed in Hermes Hybrid Recon (Hermes Medical Software), using and HOSEM reconstruction, a 3D Gaussian Filter, and applying attenuation and scatter correction. Afterwards, images were processed in Hermes Hybrid 3D (Hermes Medical Software). Area under the compensated Coefficient of Variation (CV) density curve, for CV values greater than 40% (AUC-CV40%) was calculated with the technique described by Norberg (Norberg et al., 2014). Summarily, a threshold technique was used to determine the lung volumes on the CT scan and a CV matrix (1 cm^3^ kernel) was calculated for the ventilation SPECT scan. These CV coefficients were compensated using the formula in Norberg et al. (2014). The fix point was set at *p* = 0.02% and our mode was calculated using all the studies, irrespective of the smoking status (the overall observed mode being representative of presumably normal ventilation areas). Area under the curve (AUC) was calculated on these compensated density curves for CV greater than 40% (AUC-CV40%). Initial data processing was performed while blinded to patient identity and smoking status by a member of the study team not implicated in subject recruitment.

### Statistical analysis

Normality of the data was tested using the Kolmogorov-Smirnov test. Continuous variables were reported as mean (standard deviation) or median (interquartile range) depending on the normality of their distribution. Categorical variables were reported as n (percent). Differences between variables of interest were calculated using independent t-tests, Mann-Whitney U-test or chi-squared tests, where appropriate, and one-way analysis of covariance (ANCOVA) was used to control for possible confounders.

AUC-CV40% values were compared between the two groups of subjects using independent t-tests, while correcting for age, gender and body mass index.

Relationship between AUC-CV40% values and its potential determinants (age, gender, BMI, FEV_1_, FVC, VR, CPT and D_L_CO were evaluated using Spearman correlation, linear regression analysis or binary logistic regression analysis, where applicable. Receiver operating characteristics (ROC) curve analyses were performed to compare the ability of AUC-CV40%, FEV_1_ and D_L_CO to identify subjects with higher dyspnea ratings on the mMRC scale and the presence of active smoking. In all instances, a *p*-value <0.05 was used to signify statistical significance. All analyses will be performed using SPSS v25 (IBM, Chicago, IL, United States).

## Results

30 subjects were recruited (15 in the NS group and 15 in the AS group). Demographic and clinical characteristics of the subjects are presented in Table 1. Clinical indication for the PFTs were, for the AS group: screening for COPD (n = 7), pre-operative evaluation for extra-thoracic surgery (n = 2), chronic cough (n = 2), unexplained shortness of breath (n = 4), and for the NS group: pre-operative evaluation for extra-thoracic surgery (n = 6), unexplained exertional dyspnea (n = 5), screening for lung/vascular disease in the context of collagen tissue disease (n = 2) and atypical chest pain (n = 2). Whenever available in the medical files of the subjects, chest CT scans (n = 9) and methacholine challenge testing (n = 5) revealed no significant anomaly.

**TABLE 1:** Demographic and clinical characteristics of the subjects.

	All subjects	As group	NS group	*p*-value
n	30	15	15	—
Age, *y*	59 (12)	60 (12)	57 (13)	0.61
Males, *n*	15 (50)	6 (40)	9 (60)	0.27
BMI, *kg/m* ^*2*^	29 (6)	30 (6)	27 (5)	0.15
Smoking history, *pack-years*	—	44 (24)	—	—
Comorbidities				
Hypertension, *n*	10 (33)	6 (40)	4 (27)	0.36
Diabetes, *n*	5 (17)	3 (20)	2 (13)	0.56
Coronary heart disease, *n*	3 (10)	2 (13)	1 (7)	0.50
Obstructive sleep apnea, *n*	5 (17)	4 (27)	1 (7)	0.14
Pulmonary function testing				
FEV_1_/FVC ratio	78 (5)	76 (6)	80 (5)	0.11
FEV_1_, *l*	2.95 (0.75)	2.90 (0.81)	3.00 (0.70)	0.76
FEV_1_, *z-score*	0.07 (0.96)	−0.23 (0.72)	0.39 (1.11)	0.04
FEV_1_, *%pred*	101 (14)	96 (10)	105 (16)	0.04
FVC, *l*	3.79 (0.96)	3.84 (1.15)	3.73 (0.75)	0.38
FVC, *%pred*	102 (13)	99 (11)	104 (15)	0.28
FEF_25–75%_, *%pred*	102 (29)	96 (30)	108 (27)	0.25
RV, *%pred*	96 (25)	96 (29)	96 (21)	0.94
TLC, *l*	5.79 (1.20)	5.94 (1.46)	5.63 (0.89)	0.49
TLC, *%pred*	99 (10)	98 (11)	100 (9)	0.69
DLCO, *ml/min/mmHg*	23.3 (7.2)	23.2 (9.1)	23.5 (5.1)	0.93
DLCO, *z-score*	−0.05 (1.2)	−0.12 (1.27)	0.03 (1.08)	0.74
DLCO, *%pred*	99 (19)	95 (20)	102 (18)	0.34
mMRC score	1 [1–2]	1 [1–2|	0 [0–1]	<0.001
CAT score	7 [5–11]	7 [5–13]	8 [4–10]	0.23

*p*-value refers to the statistical comparison of AS, and NS, groups. Data presented as mean (standard deviation), median [interquartile range] or n (percent) where appropriate.

BMI, body mass index; FEV_1_, forced expiratory volume in 1 second; FVC, forced vital capacity; FEF_25%–75%_, forced expiratory flow between 25% and 75% of vital capacity; RV, residual volume; TLC, total lung capacity; DLCO, diffusion capacity of the lung for carbon monoxide; mMRC, modified medical research council dyspnea scale; CAT, copd assessment test.

Despite remaining within the limits of normality, values of FEV_1_ (expressed as z-scores or percent of predicted value) were significantly lower in the AS group compared to the NS group [-0.23 (0.72) vs*.* 0.39 (1.11), *p* = 0.04 for z-score values and 96% (Bodduluri et al., 2017) vs*.* 105% (Norberg et al., 2014), *p* = 0.04 for percent-predicted values]. Other PFT variables were similar between groups, but median mMRC score was significantly higher in the AS group compared with the NS group. CAT scores were similar between the groups.

### Pulmonary regional ventilation assessment and its determinants

**FIGURE 1:**
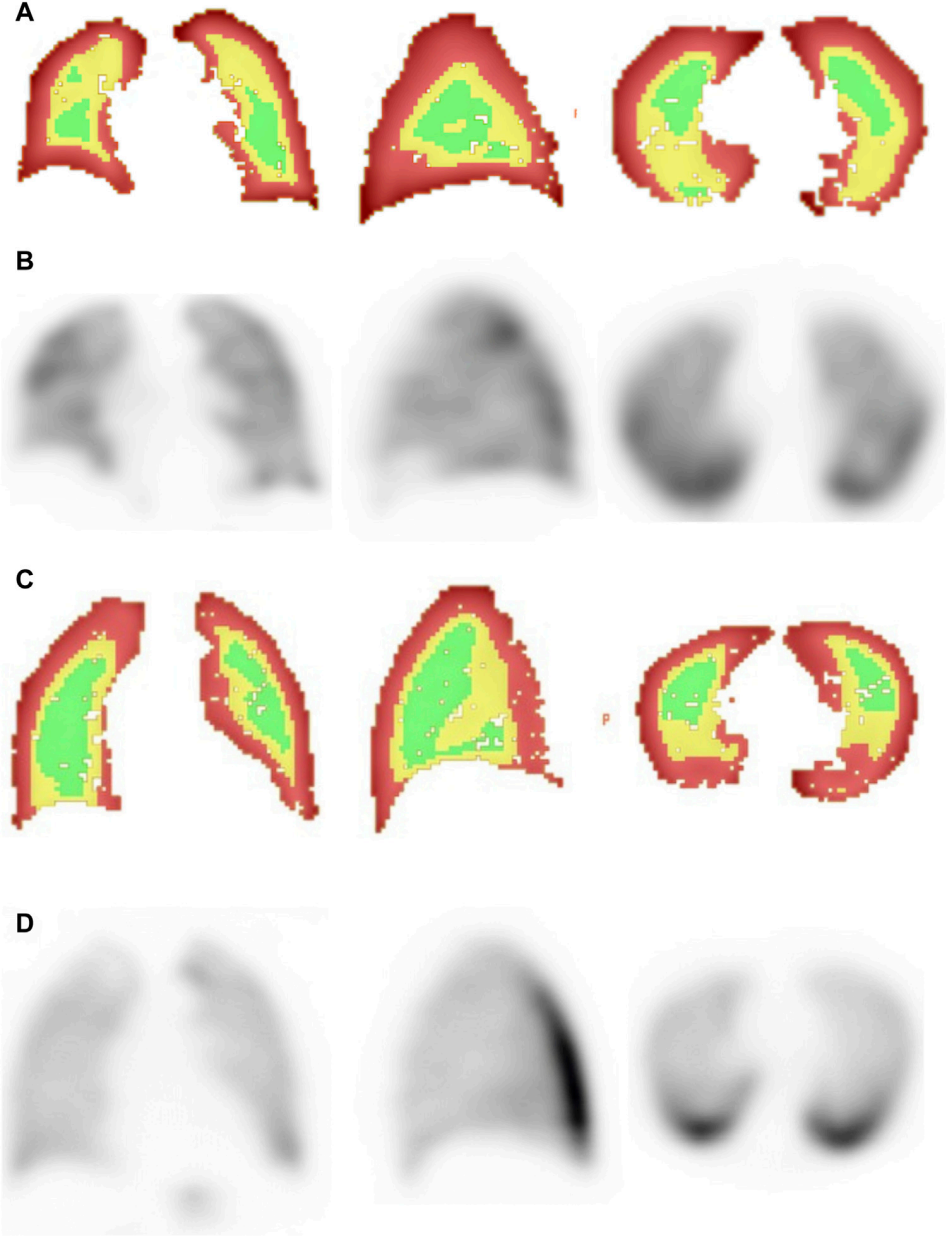
Representative images from an active smoker **(A, B)** and a never smoker **(C, D)**, showing ^99m^Tc-Technegas SPECT images in all 3 axis **(B, D)** and a 3D map of the coefficient of variation **(A, C)** where green indicates areas of low variability, yellow moderate variability, and red high variability.

**FIGURE 2:**
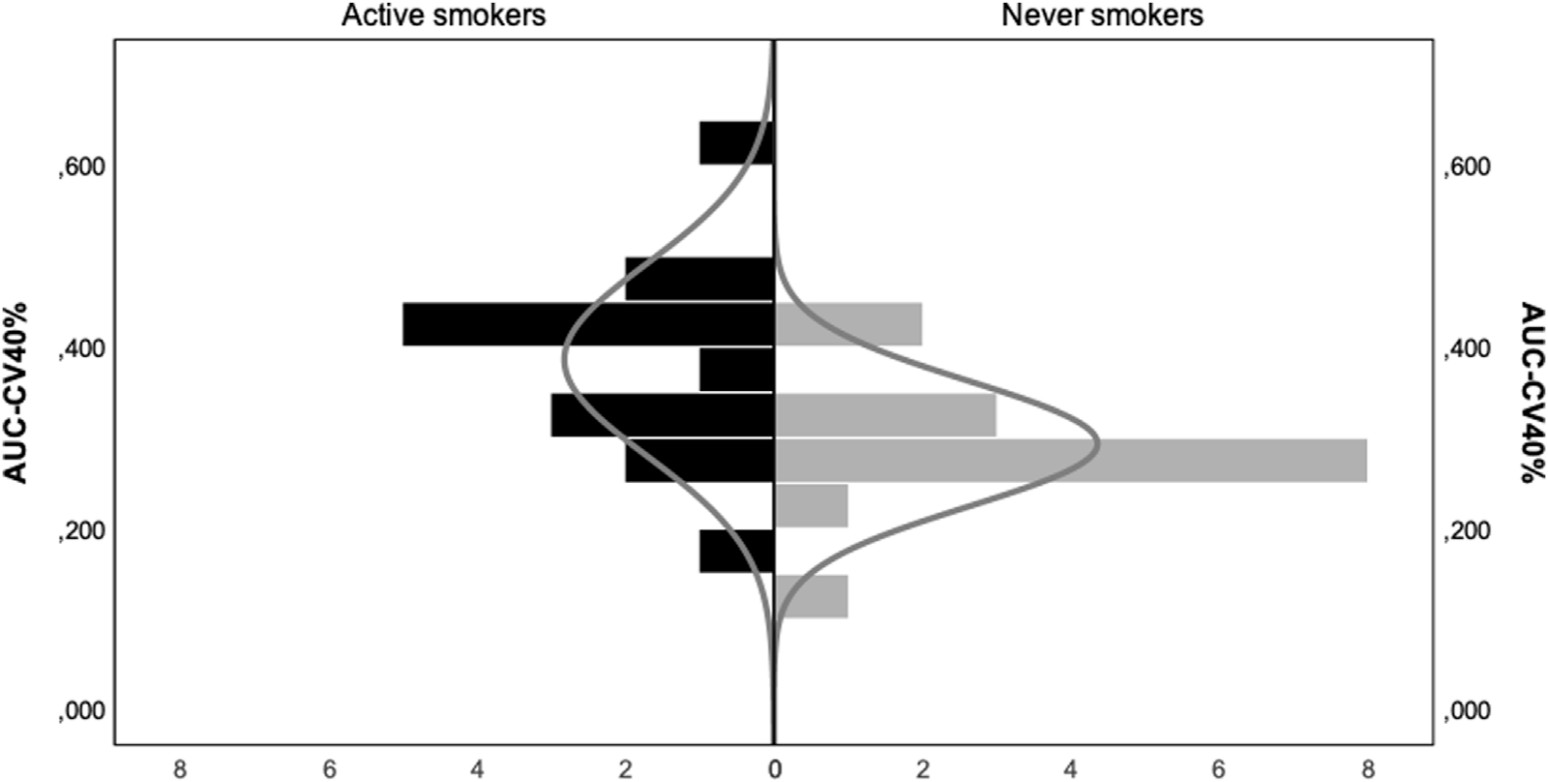
Histograms of the distributions of AUC-CV>40% values according to smoking status. Normal gaussian curves are superimposed on each distribution for reference.

Figure 1 displays a comparative example of CV distributions values in an active smoker (panels A and B) and a never smoker (panels C and D). CV were compensated using a set point of 5.93 at *p* = 0.02% and a mode of 12.6. The value of AUC-CV40% was significantly higher in the AS group compared to the NS group (0.386 ± 0.106 vs*.* 0.293 ± 0.069, *p* = 0.004). This difference remained statistically significant even when controlling for age, gender and BMI (F = 6.79, *p* = 0.015). Figure 2 displays a histogram of the comparative distribution of AUC-CV40% values for both study groups.

**FIGURE 3:**
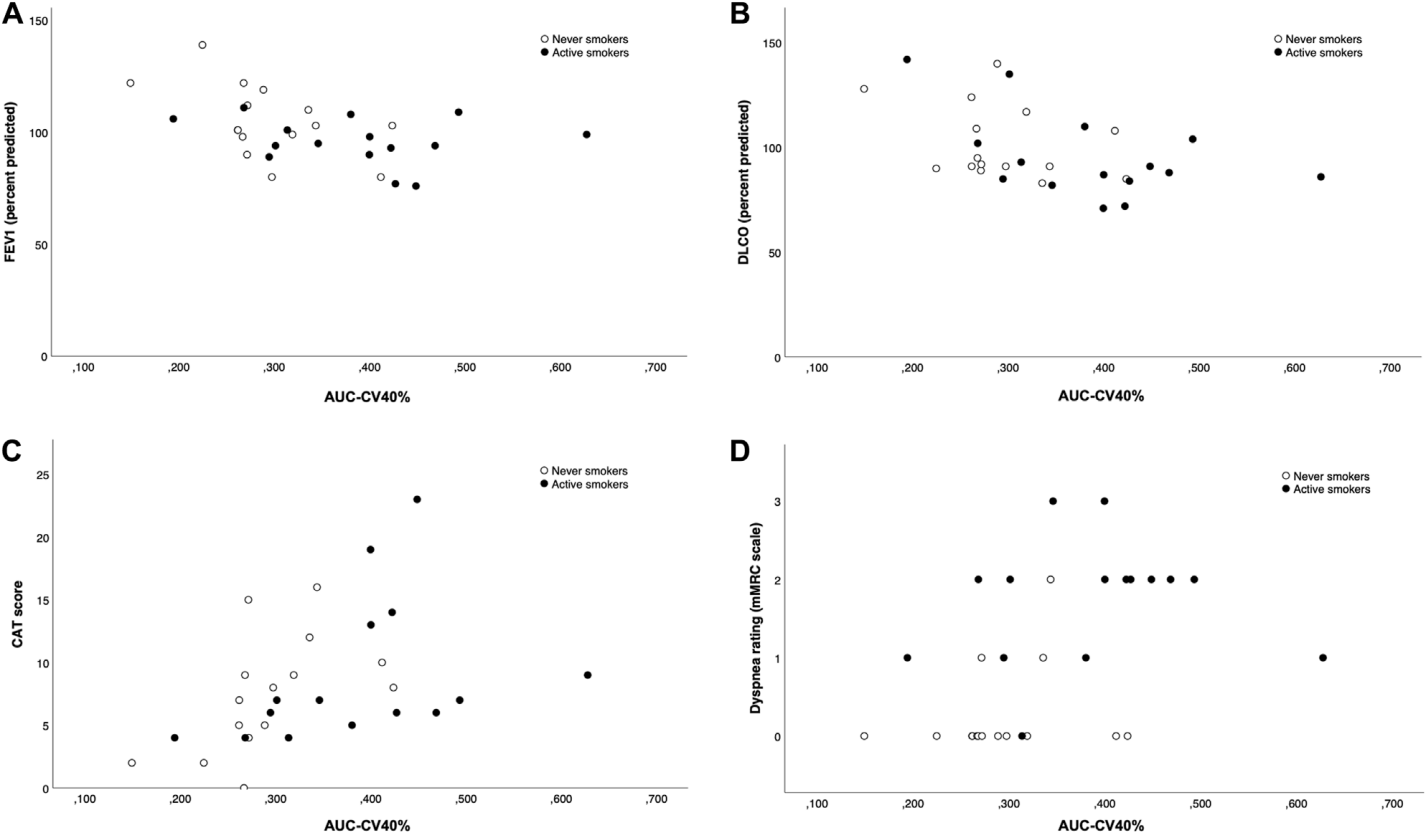
The correlation between AUC-CV>40% and FEV_1_ **(A)**, D_L_CO **(B)**, CAT score **(C)** and dyspnea rating **(D)** were all statistically significant.

There were statistically significant correlations between AUC-CV40% and FEV_1_ (rho = −0.47, *p* = 0.009), D_L_CO (rho = −0.49, *p* = 0.006), CAT score (rho = 0.55, *p* = 0.002) and mMRC score (0.54, *p* = 0.002), see Figure 3. However, the value of AUC-CV40% was not linearly correlated to cumulative smoking history (in pack-years) in the AS group (rho = 0.43, *p* = 0.11), nor to total smoking duration, FEF_25–75%_, TLC or RV (all *p* > 0.05).

**FIGURE 4:**
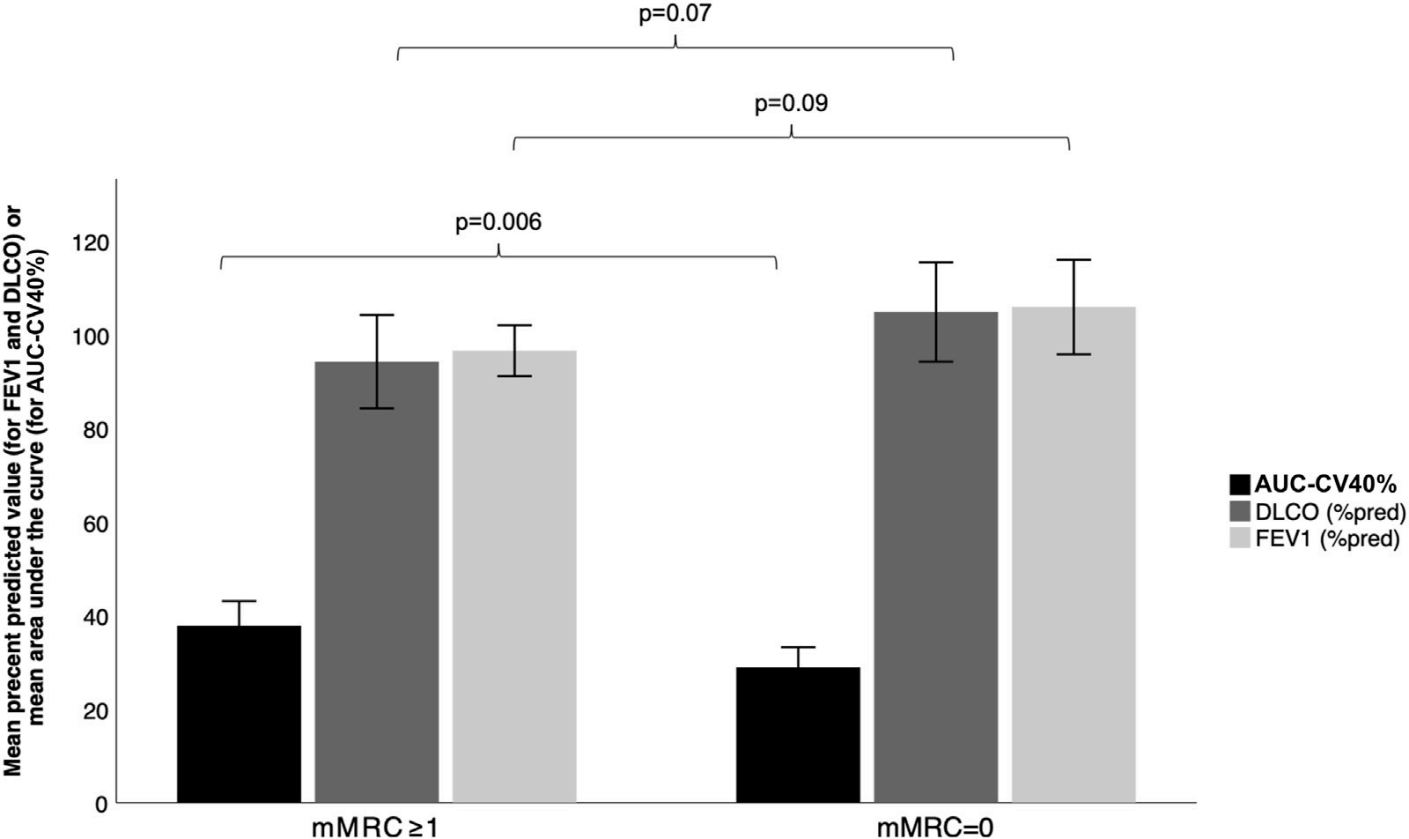
Differences in AUC-CV40%, FEV_1_ and D_L_CO between subjects according to self-reported dyspnea ratings. To simplify graphical representation of the data, values of AUC-CV40% were represented as percentage instead of decimal values (i.e., raw values were multiplied by 100). Error bars represent 95% confidence intervals.

AUC-CV40% was statistically lower in subjects with a dyspnea rating score of 0 on the mMRC scale than in those with a dyspnea rating ≥ 1 (0.289 ± 0.071 vs*.* 0.378 ± 0.102, *p* = 0.006), while FEV_1_ and D_L_CO were not different between those groups (106 ± 17 vs*.* 97 ± 11, *p* = 0.09 and 105 ± 18 vs*.* 94 ± 19, *p* = 0.07, respectively), see Figure 4.

**FIGURE 5:**
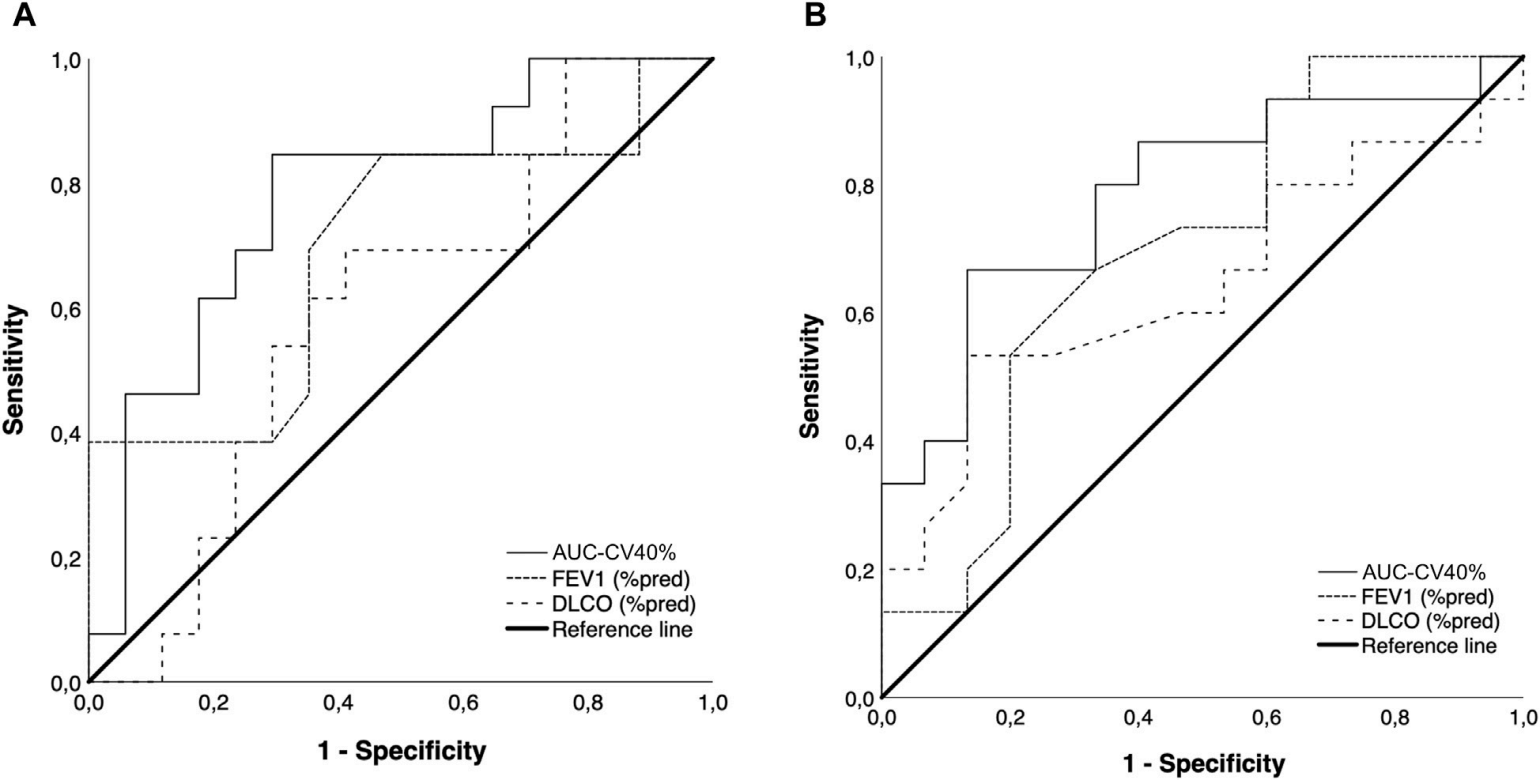
ROC curve data showing the relative performance of AUC-CV40%, FEV_1_ and D_L_CO at identifying subjects with an mMRC score ≥1 **(A)** and the presence of active smoking **(B)**. In both cases, the AUC for AUC-CV40% was higher than that of FEV_1_ and D_L_CO, and statistically significant.

A ROC analysis aiming at comparing the predictive capacity of AUC-CV>40%, FEV_1_ and D_L_CO at identifying patients with higher dyspnea levels (mMRC ≥ 1) showed that the AUC for AUC-CV40% was 0.78 (95%CI 0.61–0.95, *p* = 0.009), while that of FEV_1_ and D_L_CO were lower and did not reach statistical significance [0.69 (95%CI 0.49–0.89), *p* = 0.07 and 0.59 (95%CI 0.39–0.81), *p* = 0.37, respectively] (Figure 5, Panel A). ROC data analysis showed that an AUC-CV40% value ≥ 0.328 could identify the presence of higher dyspnea levels with a sensitivity and specificity of 85% and 71%, respectively.

A similar analysis aiming at identifying the presence of active smoking showed that the AUC for AUC-CV40% was statistically significant and higher than that of FEV_1_ and D_L_CO, which were not statistically significant [0.79 (95%CI 0.62–0.95), *p* = 0.007, 0.70 (95%CI 0.51–0.89), *p* = 0.06 and 0.64 (95%CI 0.44–0.85), *p* = 0.18, respectively]. An AUC-CV40% value of >0.345 could identify active smoking with a sensitivity and specificity of 67% and 87%, respectively (Figure 5, Panel B).

## Discussion

Our main results can be summarized as follow: 1) compared with a group of never-smoking subjects, active smokers showed significantly greater regional lung ventilation heterogeneity as measured using quantitative SPECT/CT and 2) the magnitude of these ventilatory anomalies was related to both pulmonary function tests results and relevant clinical outcomes such as exertional dyspnea and respiratory symptoms. By relating the physiological and clinical aspects of early airway disease in active smokers, our findings build on the available literature regarding the use of SPECT/CT as a tool targeting early airway disease in respiratory medicine (Norberg et al., 2013; Norberg et al., 2014) and provide the first translation of its potential as a tool for clinical practice.

According to current guidelines, the operational diagnosis of COPD requires the objective demonstration of fixed airway obstruction (i.e., a post-bronchodilator FEV_1_/FVC ratio of < 0.70) (Global Initiative for Chronic Obstructive Lung Disease). Although such a criterion is necessary to achieve a sufficient diagnostic sensitivity and specificity to allow meaningful clinical management and epidemiological characterization, the development of the disease occurs in a longitudinal and progressive fashion, in which the accelerated decline of FEV_1_ relative to FVC in smokers may cause clinical consequences well before the occurrence of ‘‘overt’’ airway obstruction. As such, respiratory symptoms in active smokers may represent early manifestation of the disease even in the face of normal FEV_1_, FVC or DLCO values (Fletcher and Peto, 1977; Jones et al., 2014; Postma et al., 2015; Vestbo and Lange, 2016; Martinez et al., 2018). From a clinical perspective, the presence of normal PFTs could lead to a false sense of security in both patients and physicians, and impair or delay the identification of patients that may be at higher risk of developing permanent consequences of tobacco smoking (Fletcher and Peto, 1977; Vestbo and Lange, 2016). The subjects in our study were specifically selected on the basis of the apparent normality of their PFTs results, but active smokers nonetheless had significantly lower values of FEV_1_ (but not D_L_CO) than non-smokers, highlighting the presence of an accelerated decline in lung function.

Our finding of a measurable and statistically significant higher degree of ventilatory inhomogeneity in active smokers using SPECT/CT provides novel data relative to the accuracy of the technique in this context and to its potential as a relevant clinical marker in clinical practice. In particular, the high specificity of AUC-CV40% for the identification of active smoking in our subjects strengthens the relationship between the observed anomalies in lung ventilation and the deleterious effects of tobacco smoking. Norberg et al. previously reported on the use of the CV threshold technique to quantify lung ventilation in healthy subjects using SPECT/CT. In addition of showing that AUC-CV20% values were related to indices of lung function testing and anthropometric variables such as age and height, they also reported that a subgroup of subjects had abnormally high AUC-CV20% values despite normal lung function testing. However, the relatively small sample size in this study did not allow for a meaningful delineation of the determinants of this subgroup. Our results confirm the significant relationships between AUC-CV and FEV_1_ and D_L_CO in a larger and well-defined population of subjects and provides a clearer relationship between smoking and the observed ventilatory anomalies. We used a different AUC-CV threshold (40%) than Norberg et al. (20%) to account for the differences in our technique (different cameras, acquisition protocols and reconstruction protocols). The threshold used by Norberg was somewhat arbitrary, based on the intersection of two patients (with different pathologies) with the mean curve of healthy subjects. They opted for this threshold as it resulted in a favorably larger range. Similarly, we adjusted our threshold so the range was favorable for further analysis. Moreover, the AUC40 for our non-smoker patients (0.293) is very similar to the AUC20 of the normal subjects Norberg used for their mean curve (0.30). More research is required on how to optimally select the AUC threshold and compensate the CV. However, our results were not particularly sensitive to these parameters.

Of note, although we did observe statistically significant differences in AUC-CV40% between the AS and NS groups, we did not find a statistically significant ‘‘dose-response’’ correlation between AUC-CV40% values and the total smoking history or total smoking duration. We hypothesize that this may be related to the well-described highly variable inter-individual intrinsic sensitivity to the effects of tobacco smoking. The representation of smoking history as pack-years is imperfect, as various smoking patterns (inhalation technique, number of cigarettes smoked per day and total length of active smoking duration), coupled to varying degrees of individual sensitivity to the effects of tobacco inhalation can results in a wide spectrum of overall toxicity and lung damage (Medici et al., 1985; Aguayo, 1994; Ingebrigtsen et al., 2010).

One of the main findings of our study is the relationship observed between AUC-CV40% values and clinical respiratory symptoms. Subjects in the AS group displayed significantly higher levels of exertional dyspnea and respiratory symptoms than those in the NS group, which is consistent with the literature describing the high prevalence of respiratory complaints and quality of life impairment in active smokers (Regan et al., 2015; Furlanetto et al., 2014; Vozoris and O’Donnell, 2015). Although the causative mechanisms underlying these symptoms in smokers are likely multifactorial and remain incompletely understood, there is evidence supporting the contribution of ventilatory anomalies in the clinical manifestation of these symptoms. In particular, abnormal lung mechanics and peripheral airway dysfunction undetectable on spirometry are thought to result in increased work of breathing and an abnormally high fractional neural drive to breathe to the respiratory muscles, which is subjectively perceived as dyspnea by patients (Elbehairy et al., 2016). In line with this, most of our subjects from the AS group were referred for PFTs because of unexplained respiratory symptoms. We observed significant correlations between the magnitude of ventilatory inhomogeneity and the level of exertional dyspnea (assessed using the mMRC scale) and respiratory symptoms (CAT score). In addition, AUC-CV40% values were significantly higher in subjects with elevated mMRC score compared to those without abnormal dyspnea, while FEV_1_ and D_L_CO values remained similar, supporting the use of AUC-CV40% not only as a marker of peripheral airway disease, but also as a clinical marker of respiratory symptoms. Together, these characteristics could position the use of AUC-CV40% as an adjunctive tool for clinicians in the evaluation of smokers with respiratory symptoms, which could help explain subjective clinical presentations, serve as an additional objective argument in patient-physician discussions regarding smoking cessation and, eventually, as a marker of risk stratification or response to interventions. These possibilities remain to be formally tested in dedicated studies.

Several limitations to the study must also be acknowledged. First, although we took several steps to exclude patients with concurrent respiratory diseases, the possibility that some subjects were included while having latent or undiagnosed asthma must be acknowledged, as its presence may not be obvious on resting pulmonary function testing. However, we specifically excluded all subjects with any clinical history of asthma, airway hyperresponsiveness and/or with abnormal methacholine challenge testing or significant bronchodilator response on spirometry. Second, we chose to evaluate respiratory symptoms using the CAT questionnaire, which was initially designed and validated in patients with a confirmed diagnosis of COPD, rather than in active smokers or healthy subjects. Although this may impair the interpretation of a subject’s given CAT score, we believe that in the context of our study the CAT questionnaire could still provide a relevant, global estimation of the burden of respiratory symptoms in our subjects. Future studies could evaluate the relationship between variables such as AUC-CV40% and more exhaustive quality of life questionnaires such as the St. George’s Respiratory Questionnaire. Third, our methodology does not allow for a mechanistic exploration of the causal relationship between AUC-CV40% and clinical symptoms. Based on our hypothesis that the higher levels of exertional dyspnea observed in the AS group may be causally related to the increased levels of ventilatory inhomogeneity via a deterioration of respiratory mechanics and increased drive to breathe, future studies should focus on the evaluation of airway resistance and neural drive activation levels using dedicated techniques. Fourth, our study was designed as a punctual exploration of the relationships between lung function, respiratory symptoms and ventilation homogeneity, precluding any conclusion regarding the potential of AUC-CV40% to act as risk predictor for the eventual development of overt COPD. A longitudinal study investigating this important question would further improve on our results and provide additional insight on the potential usefulness of SPECT/CT in clinical practice in this context. Fourth, the selection of our subjects from patients being referred for PFTs can have introduced a selection bias (towards subjects with a higher burden of symptoms and/or a higher clinical suspicion of disease). Finally, although we present the largest study investigating SPECT/CT as a marker of disease in a clinical population, our sample size remains limited and can have decreased statistical power.

## Conclusion

The increase in lung ventilatory inhomogeneities associated with active tobacco smoking can be quantified using ventilation SPECT/CT with Technegas, even in the presence of normal pulmonary function testing. As an objective estimate of these anomalies, AUC-CV40% was related to clinically relevant outcomes such as exertional dyspnea and respiratory symptoms, suggesting a potential role for this marker in the clinical evaluation of symptomatic smokers. Future studies are required to better delineate the role of ventilation SPECT/CT as a marker of response to interventions or as a prognostic indicator in this population.

## Data Availability

The raw data supporting the conclusions of this article will be made available by the authors, without undue reservation.
